# Multi-Residue Analysis of Pesticide Residues in Crude Pollens by UPLC-MS/MS

**DOI:** 10.3390/molecules21121652

**Published:** 2016-12-01

**Authors:** Zhou Tong, Yan-Can Wu, Qiong-Qiong Liu, Yan-Hong Shi, Li-Jun Zhou, Zhen-Yu Liu, Lin-Sheng Yu, Hai-Qun Cao

**Affiliations:** 1 School of Plant Protection, Anhui Agricultural University, Hefei 230036, China; tongzhou0520@163.com (Z.T.); wuyancan1989@163.com (Y.-C.W.); ahbb1104@163.com (Q.-Q.L.); shiyh@ahau.edu.cn (Y.-H.S.); ljzhou0720@163.com (L.-J.Z.); zliu@ahau.edu.cn (Z.-Y.L.); yulinsheng@ahau.edu.cn (L.-S.Y.); 2 Hefei Testing and Inspection Center for Agricultural Products Quality, Hefei 230601, China

**Keywords:** pollen, multi-residue, pesticide, UPLC-MS/MS

## Abstract

A multi-residue method for the determination of 54 pesticide residues in pollens has been developed and validated. The proposed method was applied to the analysis of 48 crude pollen samples collected from eight provinces of China. The recovery of analytes ranged from 60% to 136% with relative standard deviations (RSDs) below 30%. Of the 54 targeted compounds, 19 pesticides were detected. The major detection rates of each compound were 77.1% for carbendazim, 58.3% for fenpropathrin, 56.3% for chlorpyrifos, 50.0% for fluvalinate, 31.3% for chlorbenzuron, and 29.2% for triadimefon in crude pollen samples. The maximum values of each pesticide were 4516 ng/g for carbendazim, 162.8 ng/g for fenpropathrin, 176.6 ng/g for chlorpyrifos, 316.2 ng/g for fluvalinate, 437.2 ng/g for chlorbenzuron, 79.00 ng/g for triadimefon, and so on. This study provides basis for the research on the risks to honeybee health.

## 1. Introduction

Honeybee, belonging to the insect order Hymenoptera [1], is an important pollinator for natural ecosystems and agricultural crops [2]. Today, more than 75% of crop species worldwide—including oil crops, fruits, and vegetables—benefit from insect pollination. Farmers generally rely on the honeybee for providing food production, worth approximately 200 billion U.S. dollars [3]. Meanwhile, a great deal of hive products produced by the honeybee supply humans with various sources of nutrition.

However, a very recent phenomenon of colony collapse disorder (CCD), involving the sudden and massive disappearance of bee colonies around the world, is worrisome [4]. Within the past several years, due to global decline in honeybee population, honeybee health is a matter of public concern [4,5,6]. Since 2006, a large number of colonies have vanished. This phenomenon first emerged in North America. In Europe, the disappearance of the major honeybee colonies occurring in the vicinity of fields sprayed with pesticides was reported in 2012 [7]. CCD was reported in China in 2007 and rapidly spread nationwide. In addition, since 2007, CCD has been suspected to be occurring in Taiwan [8]. According to statistics, the number of colonies have reduced from 7.5 million in the 1990s to 6.8 million. The cause of CCD remains unknown, but there is an agreement among investigators that the possible cause of CCD could be several interacting factors [9]. The leading hypothesis for CCD links sublethal exposure to pesticides and other environmental factors, including parasitic infections and habitat loss, to honeybee losses and pollinator declines in general [10,11,12]. It is noteworthy that the pesticide-related hypothesis has received considerable attention since the emergence of CCD in 2006 [13]. Honeybees are exposed to pesticides in two ways, including foraging and contacting. Honeybees forage in an extensive range, leading them to contact contaminated food containing pollen, nectar, water, beebread, and so on. Consequently, it is particularly essential to investigate pesticide residues in pollens.

To date, a few large multi-residue methods have been developed for the determination of pesticide residues in pollens [9,14,15,16,17,18,19,20]. For example, in 2014, more than 115 pesticides were analyzed in bee pollen by LC-ESI-MS/MS. A sensitive and efficient method for routine pesticide multi-residue analysis in pollen was reported in 2015. The QuEChERS (quick, easy, cheap, effective, rugged, and safe) method was designed and successfully used for the detection of 80 environmental contaminants in pollens, analysed by LC-MS and GC-MS. Two other multi-residue methods, zirconium-based sorbents (Z-Sep) and gel permeation chromatography (GPC), determined by GC-MS, were used in the analysis of 18 pesticides in pollen in 2015. Applications of these methods resulted in crucial information about the magnitude of pesticide contamination in those pollens, particularly in North America and Europe. As a large agricultural country, China faces a high risk of threat to honeybee health because of the wide application of pesticides used for plant protection. However, up to now, there has been no single report on the level of pesticide residue in crude pollens gathered in China. The present study aimed to analyze 48 crude pollen samples, which were collected from eight provinces of China. It provides a basis for studying the risk to honeybee health.

## 2. Results and Discussion

### 2.1. Choice of Mobile Phase

Due to a wide spectrum of analyzed pesticides in a pollen matrix and great diversities between their physicochemical properties and acid–base properties, it was quite difficult to acquire a well-defined chromatographic peak and reliable liquid chromatography analysis. So that each compound could be subjected to maximum sensitivity in ultraperformance liquid chromatography (UPLC), the mobile phase composition was optimized. We conducted the test using five kinds of mobile phases for multi-residue analysis (Table 1). The results showed that the sensitivity to all compounds could be maximized at the type I of the mobile phase. 

### 2.2. Validation of the Dispersive Solid-Phase Extraction (dSPE) Clean-Up

To analyze a wide range of compounds, the multi-residue QuEChERS (quick, easy, cheap, effective, rugged, and safe) method was used for pollen samples. Analytes were extracted from the matrix by an organic solvent that was subsequently salted out from an aqueous matrix. Only a large number of proteins, aminophenols, vitamins, and lipids were present in the pollen samples, but some higher polar pigments also existed. Since the pollen matrix is complex, an additional purification step (dSPE) was necessarily used to reduce the presence of interfering substances. Initially, a dSPE step was based on primary and secondary amine-bonded silica (PSA). Since 2006, this step has been further developed. In 2006, Leandro et al. [21] used PSA and octadecyl-bonded silica (PSA/C_18_) instead of PSA-bonded silica to limit apolar interferences of the matrix. In 2010, Mullin et al. successfully adopted this method and coupled it with analysis using a dual-layer cartridge containing PSA and graphitized carbon black (GCB) to purify components from wax, pollen, bee, and beebread [22]. Here, we designed four compositions to determine the optimal clean-up conditions (Table 2) among the recoveries during this step while considering matrix effects. The test indicated that the most advantageous procedure for the dSPE clean-up is that of level B (Table 2).

### 2.3. Limits of Detection and Quantification

The method limit of detection (LOD) was defined as the lowest concentration tested in which the signal response was three times more than the background noise from the chromatogram in both transitions. The method limit of quantification (LOQ) was defined as the lowest concentration tested in which the signal response was 10 times more than the background noise from the chromatogram in the quantification transition. The ion ratio is established with the respective ratio of a standard [17]. Both LOD and LOQ values are shown in Table 3. The LOD values for all substances were below 0.5 ng/g, with the exception of aldicarb sulfoxide, which had an LOD value of 0.5291 ng/g.

### 2.4. Linearity

Linearity was evaluated by assessing the detector responses of the objective compounds from matrix-matched calibration solutions, prepared by spiking blank extracts at eight concentration levels. Since there is diversity in the signal responses between each pesticide, the range of concentrations was set at three levels. A range of eight points was used, from 5 to 200 ng/g, with the exception of 2.5–100 ng/g for carbendazim, phosphamidon, pyrimethanil, azoxystrobin, triadimefon, triazophos, and diazinon and 10–1000 ng/g for thiamethoxam, imidacloprid, iprodione, and fluvalinate. The linear ranges of all pesticides are presented in Table 4. Good linearity was observed in all cases, with correlation coefficients better than 0.9902. For all compounds studied, the signal response was linear over the range studies. Therefore, the method had a good linear relationship.

### 2.5. Matrix Effects

In this study, one of the aims was to apply the multi-residue method to a great quantity of samples to receive a summarization of environmental contamination, so standard addition calibration could not be used [23,24,25]. The matrix effect in the mass spectrometric analysis was calculated by comparing the peak areas of the standards in the mobile phase with those of the same quantities of standards, which were added to the spiked samples following the extraction. The response of each pesticide in the mobile phase was designated as the 100% response value. Table 4 shows the spectrum with matrix effects for each compound determined in pollen. The diversification with matrix effects was dependent on the physicochemical properties of the compound and the matrix. These data indicated that the concentrations of the major pesticides could be affected by the matrix effect. The external calibration curve using matrix-matched standards was an efficacious method to overcome the matrix effects when a great quantity of complex samples such as pollens are to be determined.

### 2.6. Recovery Studies

Recoveries and relative standard deviations (RSDs, measurement of precision) of the target substances were determined by spiking blank pollen samples with three different concentrations (all compounds were spiked at 5, 50, and 500 ng/g, with the exception of carbendazim, phosphamidon, pyrimethanil, azoxystrobin, triadimefon, triazophos, and diazinon, which were spiked at 2.5, 25, and 250 ng/g, and thiamethoxam, imidacloprid, iprodione, and fluvalinate were spiked at 10, 100, and 1000 ng/g) and then analyzing five replicates for three levels named low, medium, and high. Results are exhibited in Table 3. The method showed excellent performance since recoveries for the majority of the compounds were within the satisfactory range of 70%–120%. Only dichlorvos, azoxystrobin, triadimefon, chlorbenzuron, profenofos, and pyridaben had accuracies not in the acceptable range of 60%–136%. The RSD values in all cases were below 20%; in addition, when chlorbenzuron was spiked in the low range, the RSD value was more than 30%. Consequently, the procedure described in the Section 3.2 is an accurate, sensitive, and efficient method for multi-residue analysis in pollens.

### 2.7. Real Sample

**Figure 1:**
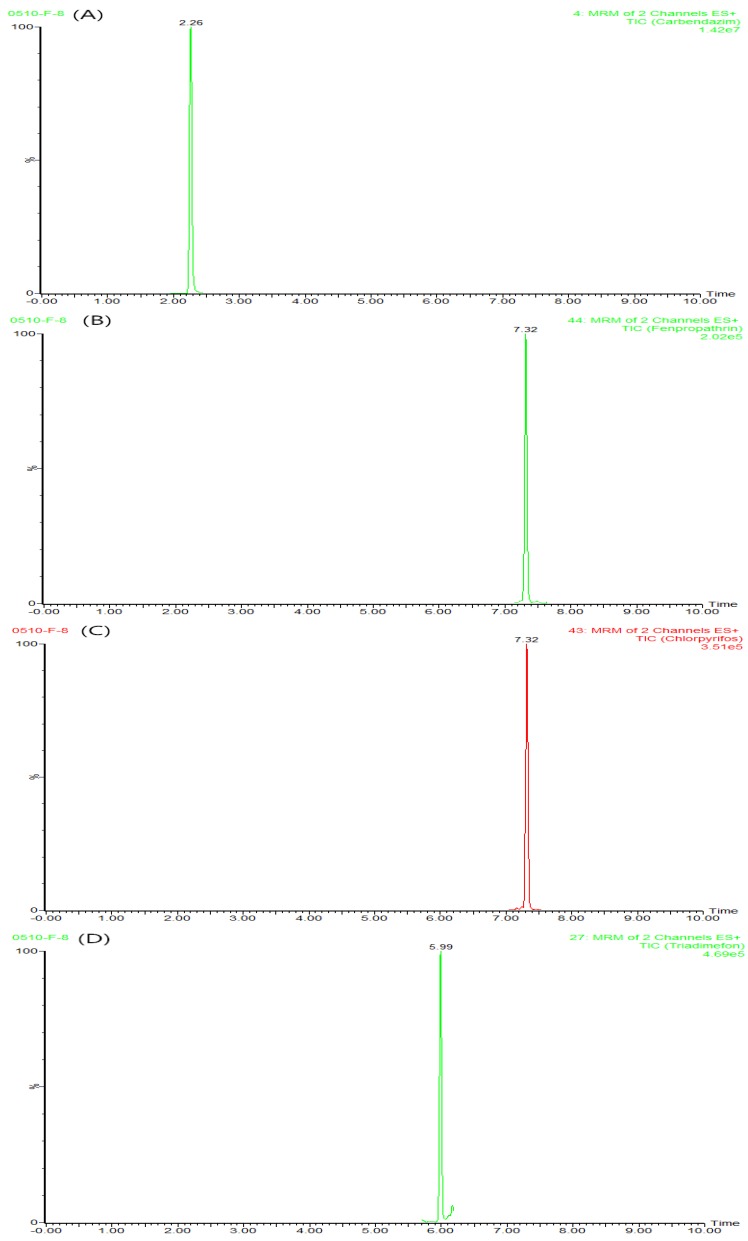
An example of the extracted Quantification ion (MRM1) chromatograms of a pollen sample, indicating the presence of (**A**) carbendazim; (**B**) fenpropathrin; (**C**) chlorpyrifos; and (**D**) triadimefon.

The multi-residue analytical method established above was applied to measure pesticide concentrations in 48 pollen samples collected from 11 apiaries in 8 provinces of China. The selected regions are characterized by agricultural events, and therefore they are prone to pollen polluted with pesticides. One hundred percent of the samples analyzed included at least one pesticide with the concentration ranging from 3.6 to 4516.4 ng/g. Of the 54 targeted compounds, 19 of them were detected. Table 5 presents the pesticides detected in 48 samples of pollen. The pesticides most commonly found in the pollens were: carbendazim (77.1%), fenpropathrin (58.3%), chlorpyrifos (56.3%), fluvalinate (50.0%), chlorbenzuron (31.3%), and triadimefon (29.2%). An emblematical chromatogram of a pollen sample including carbendazim, fenpropathrin, chlorpyrifos, and triadimefon is exhibited in Figure 1.

Since there are not any reported data about the pesticide residues in crude pollens in China, these results are interesting. As shown in Table 5, some neonicotinoid pesticides containing imidacloprid and acetamiprid were still found in pollens, but both detection rates and maximum values were of a relatively low range. This indicates that, with the increase of the reports related to the poisoning of honeybee with neonicotinoids, people use pesticides more and more cautiously during the flowering time of plants [2,3,26,27,28]. However, some pesticides highly toxic to honeybees were detected as before, such as fenpropathrin and chlorpyrifos. These would cause a huge impact on honeybees, including their behavior [29], enzyme activity [30,31,32], and so on. This suggests that the risk of pesticides highly toxic to honeybee health cannot be ignored. Thus, the investigators who focus on studying the honeybee’s health should accelerate the progress of similar researches. Meanwhile, as a compound with the highest detection rate, the maximum concentration of carbendazim reached 4516.4 ng/g. The possible reason for this is that, as a broad-spectrum pesticide for fungal disease management, carbendazim has been widely used to combat against some nectar plant diseases, including the disease caused by the fungus *Sclerotinia sclerotiorum*. Therefore, with the end of oilseed rape flowering, the detection rate of carbendazim also decreased. Currently, there are a few concerns [33] about the impact of fungicides on bees due to the absence of the acute lethal effect off fungicides on honeybees. Nevertheless, based on our results, a hypothesis that fungicides could bring some chronic effects on honeybees, including behavior, heredity, and so forth, should be proposed. The research studying how pesticides bring a high risk to honeybee health will be a long-term process.

## 3. Experimental Section

### 3.1. Chemicals and Standards

LC-grade acetonitrile and methanol were obtained from Tedia (Shanghai, China). Acetic acid, formic acid, magnesium sulfate anhydrous (MgSO_4_), and sodium acetate (NaOAc) were purchased from Sinopharm Chemical Reagent Co., Ltd. (Shanghai, China). C_18_, GCB, and PSA were obtained from Agilent Technologies (Santa Clara, CA, USA).

The compounds used in this study were selected following the requirement of Ministry of Agriculture of China (Beijing, China). All standard solutions of pesticides including methamidophos, methomyl, acephate, omethoate, carbendazim, methomyl, thiamethoxam, monocrotophos, imidacloprid, trichlorfon, dimethoate, acetamiprid, aldicarb, phosphamidon, dichlorvos, carbofuran, carbaryl, pyrimethanil, methidathion, phosmet, azoxystrobin, malathion, triadimefon, dimethomorph, triazophos, ethoprophos, iprodione, diflubenzuron, prochloraz, sulfotep, chlorbenzuron, fenthion, coumaphos, diazinon, phoxim, phorate, phosalone, difenoconazole, emamectin benzoate, profenofos, chlorpyrifos, fenpropathrin, pendimethalin, pyridaben, and fluvalinate (with purity equal than 1000 mg/L) were obtained from Agro-Environmental Protection Institute, Ministry of Agriculture of China. In addition, some pesticides with purity higher than or equal to 98.5%, including aldicarb-sulfoxide, aldicarb-sulfone, carbofuran-3-hydroxy, fenthion-sulfoxide, fenthion-sulfone, phorate-sulfoxide, phorate-sulfone, terbufos-sulfone, and terbufos-sulfoxide, were purchased from Dr. Ehrenstorfer GmbH (Augsburg, Germany). The standard stock solution of each compound at 100 mg/L was prepared in acetone or methanol, except carbendazim in dimethylformamide, and stored at −20 °C.

### 3.2. Sample Preparation

We followed QuEChERS method and made some modifications. First, 1 g of pollen samples were weighed in a 50 mL centrifuge tube, and 4 mL of water was added into the tube. The tube was shaken to blend the pollen. Then, 2 g portions of glass beads and 10 mL of 1% acetic acid mixture in acetonitrile were added and vortexed for 2 min at room temperature and for 10 min at −20 °C. Second, 0.5 g of MgSO_4_ and 2 g of NaOAc were added into each tube. The mixture was immediately hand-shaken for 1 min and centrifuged at 3500 rpm for 5 min. Subsequently, 5 mL of the acetonitrile fraction was transferred to a 15 mL centrifuge tube containing 1.25 g of the salt kits level B, described in Section 2.2. The tube was shaken by hand for 1 min and then centrifuged at 3500 rpm for 3 min. Finally, 2.5 mL of the extract was sampled in a 10 mL glass cone and was evaporated at 30 °C until dried under a gentle stream of nitrogen. Five hundred microliters of methanol was added, while the methanol layer was filtered through a 0.22 μm filter membrane and introduced into an autosampler vial for UPLC-MS/MS analysis [4,34,35,36].

### 3.3. UPLC-MS/MS Analysis

The UPLC-MS/MS instrument consists of a Waters Acquity ultraperformance liquid chromatograph (UPLC) equipped with a 1.7 μm, 2.1 mm × 100 mm particle size and Acquity BEH (ethylene bridged hybrid) C_18_ column (Waters, Milford, MA, USA) coupled to a Waters Xevo TQ triple quadrupole mass spectrometer operated in the positive electrospray ionization mode. The LC was operated under gradient conditions with mobile phases of water/methanol (98:2) + 0.05% formic acid (A) and methanol + 0.05% formic acid (B) at 40 °C [37]. It was run at 0.45 mL/min starting with 5% component B during a 0.25 min period of the injection time. Then, the composition changed to 100% component B and was maintained until 8.5 min after running. Ultimately, the mobile phase B decreased to 5% in 8.51 min and was held for 10 min to achieve re-equilibration. The total running time of analysis was 10 min. The injection volume was 3 μL.

The MS source temperature was set at 150 °C with nitrogen flow rates of 50 and 900 L/h for the cone and desolvation gases, respectively. The desolvation temperature was 500 °C. Argon was used as the collision gas with a flow of 0.15 mL/min. The mass spectrometer was operated in the multiple reaction monitoring mode (MRM) for monitoring two precursor/products ion transitions for each analyte. The target ion transition with the highest intensity (primary ion transition) was used for quantitation, whereas the second target ion transition was used for confirmation. Further confirmation was obtained through a product ion scan (PIC) for each peak, which was matched to a reference spectrum for each analyte. The quantification and confirmation calculations were determined using the software Target Lynx 4.1 (Waters Corp, Milford, MA, USA) implemented in the instrument. Ion transitions, cone voltages, collision energies, and dwell times for the analytes were shown in Table 6 [16,38].

### 3.4. Sample Collection

**Figure 2:**
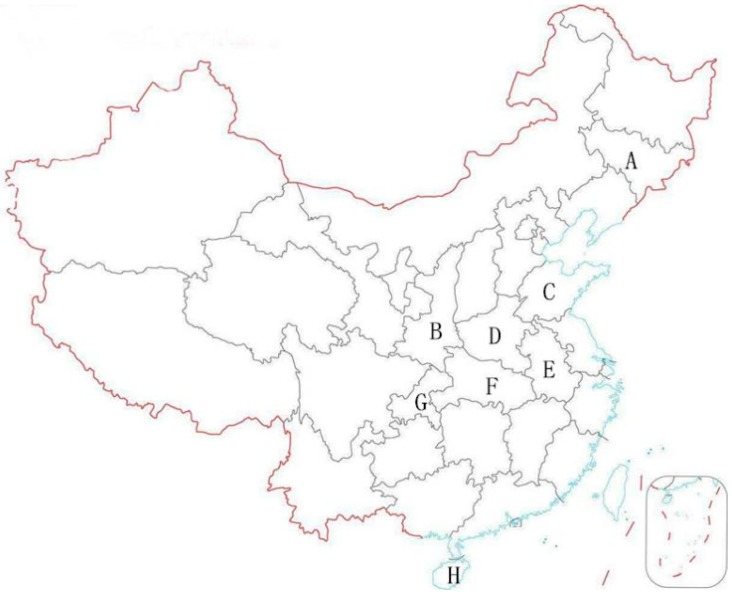
Location of the region of the sample collected in China: A—Jilin Province; B—Shanxi Province; C—Shandong Province; D—Henan Province; E—Anhui Province; F—Hubei Province; G—Chongqing Province; H—Hainan Province.

The pollen samples were provided in March, April, May, June, and July of 2016 by individual apiaries located in eight regions of China. For each month, the samples were collected in several colonies of individual apiary and repacked to obtain one crisper per apiary. All pollen samples were stored at −20 °C until further use. Figure 2 presents the location of the region of the sample collected in China.

## 4. Conclusions

In this study, an accurate and efficient modified QuEChERS protocol was established for determining 54 pesticide residues in crude pollen samples by UPLC-MS/MS analysis. More than 19 different compounds were detected in the samples collected in China. Although the maximum value of several pesticides detected in pollen is slightly lower than the LC_50_ that is reported for honeybee, the accumulation of pesticides would still endanger honeybee heath. With the appearance of the higher detection rates of carbendazim, fenpropathrin, chlorpyrifos, fluvalinate, chlorbenzuron, and triadimefon, evaluating the risk to honeybee health in the process of spraying pesticides will rely on this study. Further research on pesticide residue in other honeybee food will be necessary to better assess the risk to honeybee health.

## Figures and Tables

**Table 1:** The composition of mobile phases.

Type	Mobile Phase A	Mobile Phase B
I	water/methanol (98:2) + 0.05% formic acid	methanol + 0.05% formic acid
II	0.1% formic acid	methanol
III	0.1% formic acid	acetonitrile
IV	0.05% formic acid + 5 mmol/L ammonium acetate	methanol
V	0.05% formic acid + 5 mmol/L ammonium acetate	acetonitrile

**Table 2:** Four levels of the composition for dispersive solid-phase extraction (dSPE) clean-up.

Level	PSA	C_18_	GCB	MgSO_4_
A	50 mg	50 mg	0 mg	150 mg
B	50 mg	50 mg	3.75 mg	150 mg
C	50 mg	50 mg	7.5 mg	150 mg
D	50 mg	50 mg	15 mg	150 mg

PSA (primary secondary amine), C_18_ (octadecyl-bonded silica), GCB (graphitized carbon black).

**Table 3:** Limit of determination and quantification (LOD and LOQ) of the method, recovery of analytes, and repeatability (relative standard deviations, RSD) obtained in pollens.

Compound	LOD (ng/g)	LOQ (ng/g)	Recovery (%)			RSD (%)		
			Low (ng/g)	Medium (ng/g)	High (ng/g)	Low (ng/g)	Medium (ng/g)	High (ng/g)
Methamidophos	0.0556	0.1667	103	85.1	89.1	5.95	1.96	1.70
Acephate	0.2691	0.8072	123	87.7	91.2	12.5	1.39	1.52
Omethoate	0.1383	0.4149	112	89.6	93.5	3.75	1.79	1.93
Aldicarb-sulfoxide	0.5291	1.5873	120	93.3	97.5	3.33	3.01	2.02
Aldicarb-sulfone	0.1343	0.4030	108	98.4	98.1	3.70	0.81	2.05
Carbendazim	0.1064	0.3191	109	93.3	93.1	8.45	5.51	2.63
Methomyl	0.0337	0.1010	111	89.3	96.5	5.52	4.23	1.33
Thiamethoxam	0.0028	0.0084	125	103	101	4.63	1.78	0.71
Monocrotophos	0.0051	0.0154	112	90.9	96.9	3.57	2.21	2.68
Imidacloprid	0.0809	0.2427	92.7	85.6	96.2	8.99	1.40	3.50
Trichlorfon	0.1265	0.3794	121	86.9	98.0	3.81	3.72	3.67
Dimethoate	0.0366	0.1098	105	87.2	92.4	9.56	1.83	2.84
Carbofuran-3-hydroxy	0.0344	0.1032	94.7	90.1	99.3	6.45	1.85	2.03
Acetamiprid	0.0114	0.0343	103	86.9	97.7	5.95	1.41	2.25
Aldicarb	0.0432	0.1295	93.3	86.1	98.7	6.55	4.58	1.30
Phosphamidon	0.0037	0.0112	117	86.9	95.5	3.94	2.13	2.56
Dichlorvos	0.2483	0.7450	65.3	69.7	78.4	8.66	7.26	13.60
Carbofuran	0.0060	0.0179	88.0	87.5	95.6	4.55	4.32	1.26
Fenthion-sulfoxide	0.0202	0.0605	104	89.3	96.4	3.85	1.03	1.66
Carbaryl	0.1087	0.3261	86.7	89.1	98.1	5.33	2.89	1.25
Fenthion-sulfone	0.0369	0.1108	88.0	86.4	98.1	4.55	0.93	1.93
Pyrimethanil	0.0145	0.0435	nd	82.1	89.9	-	1.12	1.36
Phorate-sulfoxide	0.0244	0.0731	112	90.9	96.5	7.14	2.54	2.09
Phorate-sulfone	0.0241	0.0722	98.7	86.1	94.9	2.34	3.52	0.64
Methidathion	0.0095	0.0286	96.0	88.4	100	8.33	4.32	1.20
Phosmet	0.0343	0.1029	94.7	85.3	90.8	2.44	3.55	10.42
Terbufos-sulfone	0.0759	0.2277	112	93.6	98.0	6.19	3.08	2.45
Terbufos-sulfoxide	0.0243	0.0729	109	89.9	94.5	2.11	0.51	0.65
Azoxystrobin	0.0062	0.0186	131	91.7	97.1	9.35	2.66	1.90
Malathion	0.0610	0.1829	101	85.0	98.5	2.28	1.44	2.00
Triadimefon	0.0029	0.0088	133	89.6	91.2	3.46	1.79	1.52
Dimethomorph	0.0049	0.0147	70.7	85.3	93.6	6.54	0.54	1.48
Triazophos	0.0082	0.0246	112	93.3	101	7.14	6.49	1.82
Ethoprophos	0.0177	0.0530	90.7	83.5	95.3	9.18	3.08	3.81
Iprodione	0.0611	0.1833	90.0	85.5	91.4	13.6	8.66	3.83
Diflubenzuron	0.0045	0.0136	81.3	81.9	92.5	11.4	5.38	1.75
Procholraz	0.0166	0.0499	120	96.3	93.7	2.03	6.45	2.71
Sulfotep	0.0195	0.0585	89.3	87.2	93.9	5.17	2.43	4.94
Chlorbenzuron	0.0226	0.0678	68.0	89.9	92.3	30.6	6.06	1.52
Fenthion	0.0514	0.1542	86.7	91.7	88.3	13.3	2.19	0.94
Coumaphos	0.0030	0.0090	88.0	103	100	9.09	7.10	4.39
Diazinon	0.0176	0.0529	101	86.4	90.7	4.56	3.21	2.70
Phoxim	0.0135	0.0406	92.0	83.7	95.3	4.35	9.18	3.57
Phorate	0.0154	0.0462	72.0	78.7	85.6	9.62	0.59	3.99
Phosalone	0.0158	0.0475	97.3	86.7	95.6	8.55	3.24	3.16
Difenoconazole	0.0242	0.0726	104	85.9	84.4	3.85	2.34	4.52
Emamectin benzoate	0.0008	0.0025	85.3	81.1	76.5	7.16	1.14	1.09
Profenofos	0.0189	0.0568	127	92.8	84.8	4.82	8.31	1.89
Terbufos	0.1414	0.4241	97.3	77.1	86.4	8.55	5.12	1.85
Chlorpyrifos	0.0638	0.1914	103	74.7	81.9	12.5	3.76	2.78
Fenpropathrin	0.0433	0.1300	92.0	75.5	80.0	7.53	1.62	4.77
Pendimethalin	0.0236	0.0708	85.3	82.4	90.1	7.16	2.57	1.56
Pyridaben	0.0070	0.0211	136	85.3	84.8	5.88	1.08	1.89
Fluvalinate	0.0068	0.0203	98.7	76.8	66.0	1.17	9.16	2.12

**Table 4:** Matrix effects (ME), retention time (t_R_), linear range, linear regression equation, and linearity.

Compound	ME (%)	t_R_ (min)	Linear Range (ng/g)	Linear Regression Equation	Linearity
Methamidophos	−6	1.17	5–200	Y = 140.5X + 99.14	0.9984
Acephate	0	1.50	5–200	Y = 61.56X − 69.02	0.9990
Omethoate	−21	1.74	5–200	Y = 266.0X + 94.55	0.9963
Aldicarb-sulfoxide	−5	1.92	5–200	Y = 38.60X + 39.18	0.9954
Aldicarb-sulfone	−10	2.10	5–200	Y = 112.0X + 38.23	0.9994
Carbendazim	−10	2.26	2.5–100	Y = 941.6X + 11.03	0.9993
Methomyl	3	2.38	5–200	Y = 98.08X − 28.38	0.9997
Thiamethoxam	−24	2.54	10–400	Y = 66.00X + 134.2	0.9975
Monocrotophos	−20	2.67	5–200	Y = 1022X + 889.6	0.9967
Imidacloprid	−13	3.04	10–400	Y = 71.91X − 48.57	0.9996
Trichlorfon	−80	3.26	5–200	Y = 138.7X + 12.85	0.9991
Dimethoate	−76	3.29	5–200	Y = 154.1X + 79.95	0.9986
Carbofuran-3-hydroxy	−80	3.35	5–200	Y = 179.0X + 101.9	0.9991
Acetamiprid	−50	3.37	5–200	Y = 645.1X + 279.1	0.9986
Aldicarb	−24	3.97	5–200	Y = 578.5X + 620.2	0.9954
Phosphamidon	15	4.33	2.5–100	Y = 207.4X − 73.14	0.9996
Dichlorvos	−1	4.48	5–200	Y = 232.7X − 24.55	0.9998
Carbofuran	−38	4.58	5–200	Y = 854.1X − 164.8	0.9995
Fenthion-sulfoxide	−51	4.76	5–200	Y = 780.0X + 66.08	0.9999
Carbaryl	−30	4.80	5–200	Y = 139.9X + 21.77	0.9985
Fenthion-sulfone	−15	4.91	5–200	Y = 165.1X − 26.61	0.9991
Pyrimethanil	−25	5.03	2.5–100	Y = 1810X − 63.22	0.9999
Phorate-sulfoxide	−27	5.05	5–200	Y = 938.3X + 576.2	0.9990
Phorate-sulfone	−24	5.14	5–200	Y = 302.9X − 5.827	0.9998
Methidathion	−4	5.39	5–200	Y = 93.08X + 9.293	0.9998
Phosmet	−50	5.52	5–200	Y = 106.4X + 64.63	0.9976
Terbufos-sulfone	16	5.62	5–200	Y = 96.91X − 66.94	0.9975
Terbufos-sulfoxide	20	5.64	5–200	Y = 192.5X − 80.41	0.9983
Azoxystrobin	25	5.67	2.5–100	Y = 363.1X + 48.65	0.9990
Malathion	13	5.89	5–200	Y = 151.5X − 111.6	0.9952
Triadimefon	15	5.99	2.5–100	Y = 310.0X − 135.0	0.9955
Dimethomorph	−20	6.01	5–200	Y = 168.5X − 169.4	0.9903
Triazophos	16	6.05	2.5–100	Y = 747.9X + 45.03	0.9995
Ethoprophos	−1	6.19	5–200	Y = 305.2X − 87.03	0.9992
Iprodione	−46	6.33	10–400	Y = 55.78X − 106.2	0.9952
Diflubenzuron	−6	6.34	5–200	Y = 86.59X − 52.92	0.9941
Prochloraz	−27	6.44	5–200	Y = 587.4X − 606.3	0.9902
Sulfotep	−33	6.44	5–200	Y = 1144X − 1042	0.9955
Chlorbenzuron	−27	6.48	5–200	Y = 89.28X − 107.6	0.9940
Fenthion	−66	6.51	5–200	Y = 101.6X − 19.60	0.9983
Coumaphos	−35	6.54	5–200	Y = 63.55X − 95.86	0.9914
Diazinon	9	6.55	2.5–100	Y = 1220X − 596.6	0.9907
Phoxim	−20	6.63	5–200	Y = 49.86X + 5.412	0.9969
Phorate	−12	6.67	5–200	Y = 33.04X − 65.92	0.9996
Phosalone	−26	6.69	5–200	Y = 49.75X − 34.82	0.9944
Difenoconazole	−10	6.84	5–200	Y = 520.7X − 417.0	0.9949
Emamectin benzoate	−12	6.83	5–200	Y = 1177X − 804.8	0.9931
Profenofos	−25	7.05	5–200	Y = 182.7X − 171.3	0.9935
Terbufos	−34	7.10	5–200	Y = 83.82X − 1.636	0.9989
Chlorpyrifos	−44	7.32	5–200	Y = 327.6X + 67.46	0.9995
Fenpropathrin	−46	7.32	5–200	Y = 407.6X + 67.03	0.9999
Pendimethalin	−26	7.34	5–200	Y = 230.1X − 83.93	0.9987
Pyridaben	−73	7.67	5–200	Y = 1811X − 172.8	0.9984
Fluvalinate	−77	7.74	10–400	Y = 472.3X + 334.3	0.9997

**Table 5:** Several typical pesticides in pollen samples.

Compound	Positive Sample	Total Number of Sample	Detection Rate (%)	Detected Concentration Ranges (ng/g)	Max Value (ng/g)	Central Values (ng/g)
carbendazim	37	48	77.1	3.200–4516	4516	44.00
fenpropathrin	28	48	58.3	5.000–162.8	162.8	21.70
chlorpyrifos	27	48	56.3	5.000–176.6	176.6	23.60
fluvalinate	27	48	50.0	6.600–316.2	316.2	33.00
chlorbenzuron	15	48	31.3	5.000–437.2	437.2	27.00
triadimefon	14	48	29.2	2.600–79.00	79.00	19.70
acetamiprid	8	48	1.7	5.200–63.60	63.60	8.300
imidacloprid	7	48	1.5	17.60–49.80	49.80	27.60

**Table 6:** Ion transitions used for the quantification (MRM1) and confirmation (MRM2), dwell time, cone voltages, and collision energies for MS.

Compound	Transitions	Dwell Time (s)	Cone Voltage (V)	Collision Energy (eV)
Methamidophos	Quantification ion 142 > 93.9	0.050	17	13
	Confirmation ion 142 > 124.9		17	13
Acephate	Quantification ion 184.1 > 143	0.036	8	8
	Confirmation ion 184.1 > 125.1		8	18
Omethoate	Quantification ion 214.1 > 125.1	0.028	16	22
	Confirmation ion 214.1 > 183.1		16	11
Aldicarb sulfoxide	Quantification ion 207 > 89	0.028	13	14
	Confirmation ion 207 > 132		13	10
Aldicarb sulfone	Quantification ion 223 > 86	0.028	22	14
	Confirmation ion 223 > 148		22	10
Carbendazim	Quantification ion 192.1 > 160.1	0.028	24	18
	Confirmation ion 192.1 > 132.1		24	28
Methomyl	Quantification ion 163 > 88	0.028	17	10
	Confirmation ion 163 > 106		17	10
Thiamethoxam	Quantification ion 292.1 > 210.9	0.044	18	12
	Confirmation ion 292.1 > 181		18	24
Monocrotophos	Quantification ion 224.1 > 127.1	0.044	15	16
	Confirmation ion 224.1 > 98.1		15	12
Imidacloprid	Quantification ion 256.1 > 209.1	0.028	23	15
	Confirmation ion 256.1 > 175.1		23	20
Trichlorfon	Quantification ion 257 > 109	0.028	22	18
	Confirmation ion 257 > 79		22	30
Dimethoate	Quantification ion 230.1 > 199	0.028	12	10
	Confirmation ion 230.1 > 125		12	20
Carbofuran-3-hydroxy	Quantification ion 238 > 163	0.028	25	16
	Confirmation ion 238 > 181		25	10
Acetamiprid	Quantification ion 223 > 126	0.028	23	20
	Confirmation ion 223 > 56.1		23	15
Aldicarb	Quantification ion 212.8 > 88.9	0.078	20	16
	Confirmation ion 212.8 > 115.9		20	12
Phosphamidon	Quantification ion 300.1 > 174.1	0.028	17	14
	Confirmation ion 300.1 > 127.1		17	25
Dichlorvos	Quantification ion 221 > 109	0.022	23	22
	Confirmation ion 221 > 79		23	34
Carbofuran	Quantification ion 222.1 > 165.1	0.022	25	16
	Confirmation ion 222.1 > 123		25	16
Fenthion-sulfoxide	Quantification ion 295 > 109	0.022	29	32
	Confirmation ion 295 > 280		29	18
Carbaryl	Quantification ion 202 > 145	0.022	19	22
	Confirmation ion 202 > 117		19	28
Fenthion-sulfone	Quantification ion 311 > 125	0.022	29	22
	Confirmation ion 311 > 109		29	28
Pyrimethanil	Quantification ion 200.2 > 107	0.022	42	24
	Confirmation ion 200.2 > 82		42	24
Phorate-sulfoxide	Quantification ion 277 > 96.9	0.022	15	32
	Confirmation ion 277 > 143		15	20
Phorate-sulfone	Quantification ion 293 > 96.9	0.022	15	30
	Confirmation ion 293 > 115		15	24
Methidathion	Quantification ion 303 > 145	0.022	10	10
	Confirmation ion 303 > 85.1		10	20
Phosmet	Quantification ion 318 > 160	0.018	20	14
	Confirmation ion 340 > 214.1		30	14
Terbufos-sulfone	Quantification ion 321.2 > 171	0.018	19	12
	Confirmation ion 321.2 > 97		19	40
Terbufos-sulfoxide	Quantification ion 305 > 187	0.018	10	11
	Confirmation ion 305 > 97		10	40
Azoxystrobin	Quantification ion 404 > 372	0.017	17	15
	Confirmation ion 404 > 329		17	30
Malathion	Quantification ion 331 > 127	0.018	18	12
	Confirmation ion 331 > 79		18	40
Triadimefon	Quantification ion 294.1 > 197.2	0.018	22	15
	Confirmation ion 294.1 > 69.3		22	20
Dimethomorph	Quantification ion 388.1 > 300.9	0.018	30	20
	Confirmation ion 388.1 > 165		30	30
Triazophos	Quantification ion 314.1 > 161.9	0.013	22	18
	Confirmation ion 314.1 > 118.9		22	35
Ethoprophos	Quantification ion 243.2 > 131	0.008	18	20
	Confirmation ion 243.2 > 97		18	31
Iprodione	Quantification ion 330 > 244.7	0.008	12	16
	Confirmation ion 330 > 288		12	15
Diflubenzuron	Quantification ion 310.9 > 157.9	0.008	20	14
	Confirmation ion 310.9 > 140.9		20	36
Procholraz	Quantification ion 376 > 308	0.008	20	15
	Confirmation ion 376 > 266		20	15
Sulfotep	Quantification ion 323 > 97	0.008	17	32
	Confirmation ion 323 > 171		17	15
Chlorbenzuron	Quantification ion 309 > 155.9	0.008	22	26
	Confirmation ion 309 > 138.8		22	18
Fenthion	Quantification ion 279 > 168.9	0.008	30	18
	Confirmation ion 279 > 105		30	28
Coumaphos	Quantification ion 363.1 > 307	0.008	21	16
	Confirmation ion 363.1 > 289		21	24
Diazinon	Quantification ion 305.1 > 169	0.008	20	22
	Confirmation ion 305.1 > 96.9		20	35
Phoxim	Quantification ion 299 > 129	0.008	12	13
	Confirmation ion 299 > 153		12	7
Phorate	Quantification ion 261 > 97	0.008	14	28
	Confirmation ion 261 > 75		14	10
Phosalone	Quantification ion 367.9 > 181.9	0.008	12	14
	Confirmation ion 367.9 > 110.9		12	42
Difenoconazole	Quantification ion 406 > 251.1	0.026	37	25
	Confirmation ion 406 > 111.1		37	60
Emamectin benzoate	Quantification ion 886.5 > 158.1	0.022	20	32
	Confirmation ion 886.5 > 81.9		20	64
Profenofos	Quantification ion 372.9 > 302.6	0.022	25	20
	Confirmation ion 372.9 > 127.9		25	40
Terbufos	Quantification ion 289 > 103	0.022	12	8
	Confirmation ion 289 > 57.2		12	22
Chlorpyrifos	Quantification ion 350 > 97	0.022	27	32
	Confirmation ion 350 > 198		27	20
Fenpropathrin	Quantification ion 350.1 > 97	0.022	15	34
	Confirmation ion 350.1 > 125		15	14
Pendimethalin	Quantification ion 252.2 > 212.2	0.022	12	10
	Confirmation ion 252.2 > 194.1		12	17
Pyridaben	Quantification ion 365.1 > 147.1	0.022	19	24
	Confirmation ion 365.1 > 309.1		19	12
Fluvalinate	Quantification ion 507 > 181.1	0.022	15	30
	Confirmation ion 507 > 208.1		15	12
